# Pretreatment anti-Müllerian hormone predicts for loss of ovarian function after chemotherapy for early breast cancer^☆^

**DOI:** 10.1016/j.ejca.2013.07.014

**Published:** 2013-11

**Authors:** Richard A. Anderson, Mikkel Rosendahl, Thomas W. Kelsey, David A. Cameron

**Affiliations:** a MRC Centre for Reproductive Health, University of Edinburgh, Queens Medical Research Institute, 47 Little France Crescent, Edinburgh EH16 4TJ, UK; b Laboratory of Reproductive Biology, The Juliane Marie Centre for Women, Children and Reproduction, Copenhagen University Hospital, Copenhagen, Denmark; c School of Computer Science, University of St. Andrews, St. Andrews, UK; d Edinburgh Breast Unit, Western General Hospital, Crewe Road South, Edinburgh EH4 2XU, UK; e Edinburgh University Cancer Research Centre, Western General Hospital, Crewe Road South, Edinburgh EH4 2XU, UK

**Keywords:** AMH, Ovarian reserve, Chemotherapy, Amenorrhoea, Fertility, Breast cancer

## Abstract

**Aim:**

Improving survival for women with early breast cancer (eBC) requires greater attention to the consequences of treatment, including risk to ovarian function. We have assessed whether biochemical markers of the ovarian reserve might improve prediction of chemotherapy related amenorrhoea.

**Methods:**

Women (*n* = 59, mean age 42.6 years [(range 23.3–52.5]) with eBC were recruited before any treatment. Pretreatment ovarian reserve markers (anti-Müllerian hormone [AMH], follicle-stimulating hormone [FSH], inhibin B) were analysed in relation to ovarian status at 2 years.

**Results:**

Pretreatment AMH was significantly lower in women with amenorrhoea at 2 years (4.0 ± 0.9 pmol/L versus 17.2 ± 2.5, *P* < 0.0001), but FSH and inhibin B did not differ between groups. By logistic regression, pretreatment AMH, but not age, FSH or inhibin B, was an independent predictor of ovarian status at 2 years (*P* = 0.005; odds ratio 0.013). We combined these data with a similar cohort (combined *n* = 75); receiver–operator characteristic analysis for AMH gave area under curve (AUC) of 0.90 (95% confidence interval (CI) 0.82–0.97)). A cross-validated classification tree analysis resulted in a binary classification schema with sensitivity 98.2% and specificity 80.0% for correct classification of amenorrhoea.

**Conclusion:**

Pretreatment AMH is a useful predictor of long term post chemotherapy loss of ovarian function in women with eBC, adding significantly to the only previously established individualising predictor, i.e. age. AMH measurement may assist decision-making regarding treatment options and fertility preservation procedures.

## 1 Introduction

Recent years have seen a steady improvement in the long-term survival for many malignancies, including early breast cancer (eBC).^1^ Consideration of the late effects of treatment is therefore assuming greater prominence. Chemotherapy has long been recognised to have adverse effects on ovarian function,^2–4^ although detailed understanding of the effects of chemotherapy on the ovary is less abundant.^5,6^ A survival benefit of chemotherapy-related amenorrhoea has been suggested in breast cancer,^7,8^ although the risk of amenorrhoea could reflect individual responsiveness to chemotherapy.^9^ For premenopausal women with moderate risk of eBC, there is a risk benefit assessment to be made about whether to undergo chemotherapy, and for many, potential loss of fertility/ovarian function may influence their choice of adjuvant therapies.

Age at treatment is a clearly identified risk factor for the development of amenorrhoea^3,4^ reflecting the progressive decline in the ovarian reserve.^10,11^ There is however very large variation in follicle number between women of the same age, thus there is a need for a reliable marker to allow improved individualisation of advice to women facing potentially curative cancer therapy that will significantly affect treatment decisions related to subsequent reproductive function. There is now a substantial body of evidence indicating that serum measurement of anti-Müllerian hormone (AMH) is a clinically useful biomarker of the ovarian reserve.^12–15^ It is a more accurate predictor than other hormonal markers of the ovarian reserve (follicle-stimulating hormone (FSH), inhibin B),^13,16^ and its stability across the menstrual cycle is of practical value.^17^ It is however sensitive to long-term gonadotrophin suppression e.g. by gonadotrophin-releasing hormone (GnRH) analogues^18^ and the contraceptive pill.^19^ While it is probably of similar value to ultrasound determination of antral follicle count the latter is less readily available and requires expertise to maximise accuracy.^20^ A number of studies have demonstrated that AMH is lower in women who have had cancer treatment^18,21–25^ but the predictive value of AMH for post-chemotherapy amenorrhoea is unclear.^26–28^

We have therefore carried out a prospective study to test the hypothesis that AMH, measured at the time of diagnosis, would be a clinically useful predictor of amenorrhoea after chemotherapy for eBC, in comparison to age at diagnosis or other biochemical markers of the ovarian reserve.^16^ As some women show recovery of ovarian function after chemotherapy, the primary analysis of this study was performed at two years after diagnosis.

## 2 Patients and methods

A total of 60 premenopausal women with early breast cancer were recruited to this study, between March 2007 and June 2009, in two centres (Edinburgh Breast Unit and Copenhagen University Hospital – Rigshospitalet): one woman was withdrawn as she was found to be ineligible, thus data were available for 59 women. The study received Ethics committee approval, and all women gave informed consent in writing. The design of the study was prospective, with women recruited before receiving any treatment for their breast cancer, and followed up for a total of 2 years. Inclusion criteria were primary operable breast cancer without evidence of metastases, and being premenopausal assessed by regular menses in the absence of sex steroid contraception, or premenopausal gonadotrophin and estradiol concentrations. Women were not included if they had had previous surgery to either ovary or had received chemotherapy previously. Recruitment to this study did not alter the management of their breast cancer, and women were still considered for any interventional research study for which they might also be eligible.

**Fig. 1:**
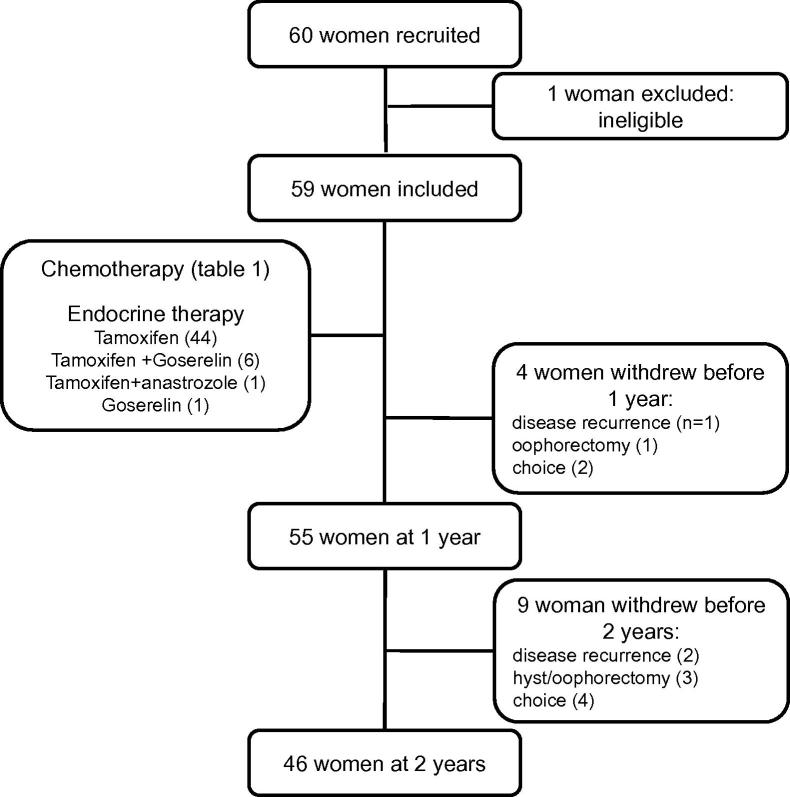
Consort diagram showing patient numbers at recruitment, at key points during the study, and reasons for withdrawal from the study.

The mean age of the women was 42.6 years (range 23.3–52.5). Of the 59 women in this study, a total of 13 withdrew before the end of the study 2 years later (Fig. 1). This was for reasons of disease recurrence in three, four had an oophorectomy and/or hysterectomy and for personal reasons in six. Data were available for analysis from 55 women at 1 year and 46 at 2 years.

Table 1 gives details of chemotherapy regimens; 44 women received tamoxifen treatment following chemotherapy, and seven received goserelin (only one woman received goserelin but not tamoxifen) and one woman was treated with anastrozole in addition to tamoxifen.

Women kept menstrual diaries throughout, with data subsequently coded as amenorrhoea when there had been no bleeding in the previous 6 months, or as having on-going menses. The primary end-point of the study was of amenorrhoea versus ongoing menses at 2 years. Blood samples were obtained pretreatment, after one and two cycles of chemotherapy, and at 1 year, and were scheduled to be in the early follicular phase (days 2–5) in women with ongoing menses.

Serum hormones were measured as previously described^18^ with the exception of AMH which was measured by the Gen II enzyme-linked immunosorbent assay (ELISA) kit (Beckman Coulter, Chaska, MN). This has a sensitivity of 0.16 ng/ml (1.1 pmol/L) and in-house intra- and inter-assay coefficient of variation of <6%.

### 2.1 Statistical analysis

Data are presented as mean ± standard error of mean (SEM), and range when specified. Spearman’s test was used to test relationships between age and AMH pretreatment and other pairs of variables. Initial analysis of predictors of amenorrhoea (i.e. the primary objective of the study) was performed by Student’s *t* test, with log transformation of hormonal data to correct for heterogeneity of variance. Because of relationships between the variables, a multivariate logistic regression analysis was performed to determine which factors independently predicted amenorrhoea. Analyses were performed using SPSS (version 20; IBM Corporation).

To improve the power of the analysis we combined this dataset in a secondary analysis with a previous very similar cohort of premenopausal women with eBC recruited with the same inclusion and exclusion criteria^18,27^ for which pretreatment AMH and amenorrhoea versus menses at 2 years was also available. In that study AMH had been measured using a different ELISA: data were converted as described elsewhere.^29^ The relative predictive importance of AMH and age in the combined cohort of 75 women was investigated using two distinct methods:

(1) Analysis of the area under curve (AUC) of receiver–operator characteristic (ROC) plots^30^ for age and AMH as separate predictors.
(2) The use of Random Forests^31^ to derive 2000 classification trees each of which uses age and AMH to predict amenorrhoea. To estimate the relative importance of age and AMH, we calculated the total decrease in node impurities (measured by the Gini index) from splitting on each variable, averaged over all trees.

We also performed a top-down induction of a classification tree^32^ using both age and AMH as potential classifiers. The induction was done in two stages. We first derived the classification tree by recursive identification of the predictor variable that splits the data into two groups, so that the tradeoff between sensitivity and specificity is optimal. We then performed a 10-fold cross-validation calculation to prune the full tree in order to minimise the error rate when generalised to unseen observations, and converted it into a classification mosaic chart. The ROC, Random Forest and classification tree analyses were performed using R (version 2.15.1, The R Foundation for Statistical Computing).

## 3 Results

**Fig. 2:**
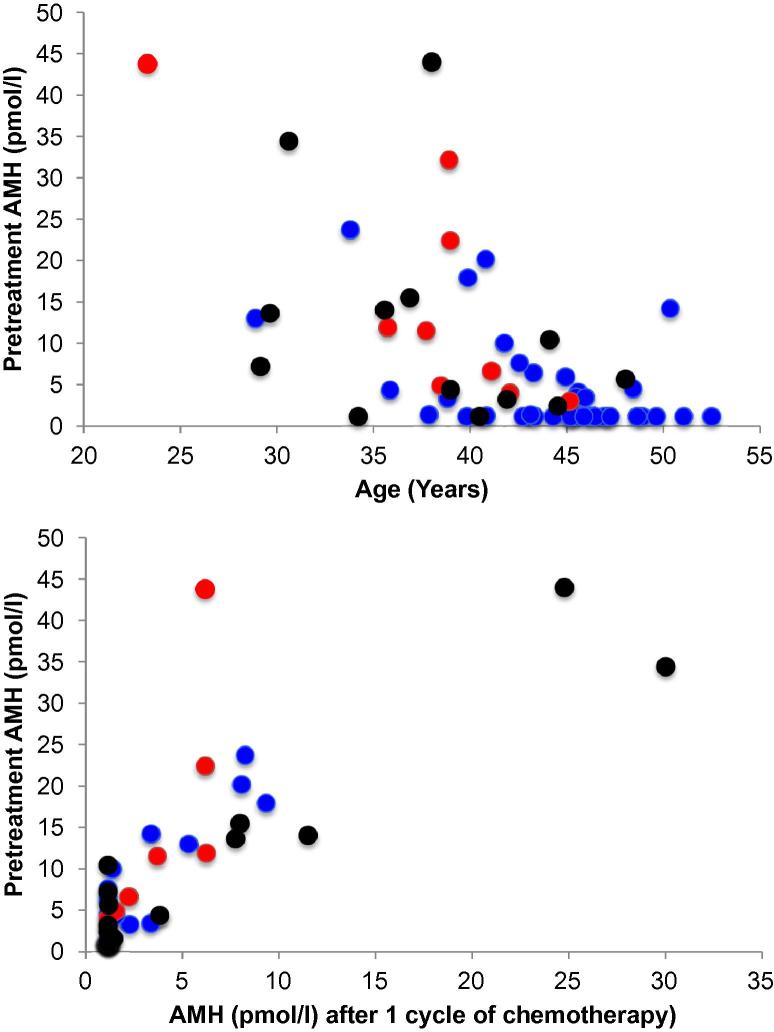
(A) Pretreament anti-Müllerian hormone (AMH) versus age in women with newly-diagnosed early breast cancer (*n* = 59). Spearman rho = −0.56, *P* < 0.0001. (B) Relationship between AMH pretreatment and after one cycle of chemotherapy (Spearman rho = 0.76, *P* < 0.0001). Red symbols indicate women subsequently demonstrated to have ongoing menses at 2 years, blue, those with amenorrhoea at that time, black, those who withdrew from the study before 2 years.

At pretreatment, there was an inverse relationship between age and serum AMH (Spearman rho = −0.56, *P* < 0.0001; Fig. 2a). AHM fell during chemotherapy, from 7.9 ± 1.3 pmol/L pretreatment to 3.5 ± 0.7 pmol/L after one cycle (*P* < 0.001). There was a significant relationship between pretreatment AMH and that after the first cycle of chemotherapy (rho = 0.76, *P* < 0.0001; Fig. 2b), indicating that after one cycle of chemotherapy AMH remained higher in women with a higher pretreatment AMH. However after two or more cycles and at 1 year, AMH was undetectable or close to the limit of detection in all women. To test whether younger women might have received lower doses of cyclophosphamide, the relationship between age and dose (total dose received in mg/m^2^) was calculated. There was no relationship between age and dose of cyclophosphamide (*P* = 0.57).

The primary objective of this study was the assessment of pretreatment AMH in comparison with other markers of the ovarian reserve as a predictor of post-chemotherapy ovarian function, using amenorrhoea at 2 years as an indicator of absent ovarian activity. We have previously robustly validated this using a full panel of endocrine and ultrasound markers,^27^ confirmed by the present data as serum estradiol was significantly lower in women with amenorrhoea versus ongoing menses (91 ± 19 versus 302 ± 143 pmol/L, *P* = 0.001).

**Fig. 3:**
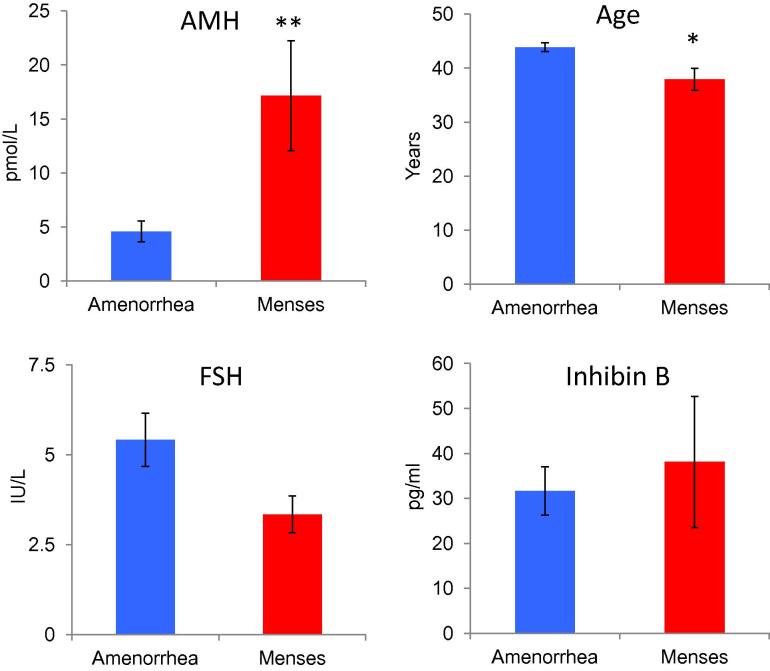
Pretreatment concentrations of anti-Müllerian hormone (AMH), follicle-stimulating hormone (FSH) and inhibin B and age by the presence of amenorrhoea or ongoing menses at 2 years. Mean ± standard error of mean (SEM), *n* = 37 and *n* = 9 respectively. ^∗^*P* = 0.004; ^∗∗^*P* < 0.0001.

At 2 years, 30 women were amenorrhoeic and nine had ongoing menses (after excluding women taking goserelin). Pretreatment AMH showed a significant positive correlation with menses; women with low pretreatment AMH were more prone to be amenorrhoeic at 2 years (Fig. 3a; Table 2). Age at diagnosis was also significantly different between these groups, being higher in those developing amenorrhoea, but pretreatment FSH and inhibin B were not significantly different (Fig. 3b–d). At 1 year, 45 women were amenorrhoeic whereas 10 had ongoing menses. Similar results were obtained to those seen at 2 years (Table 2), with mean pre-treatment AMH concentrations lower in amenorrhoeic women. Age was also significantly different but FSH and inhibin B were again not different. These data therefore indicate that both pre-treatment AMH and age are predictors of amenorrhoea at both post-treatment time points analysed.

As FSH and inhibin B are established markers of the ovarian reserve,^16^ logistic regression was used to investigate which variables have independent predictive value. Age and pretreatment concentrations of AMH, FSH and inhibin B were included in the analysis. Only AMH remained a significant predictor of amenorrhoea at 24 months (*P* = 0.005) with odds ratio 0.013 (95% confidence interval (CI) 0.001–0.227). Age, FSH and inhibin B were not significant predictors.

**Fig. 4:**
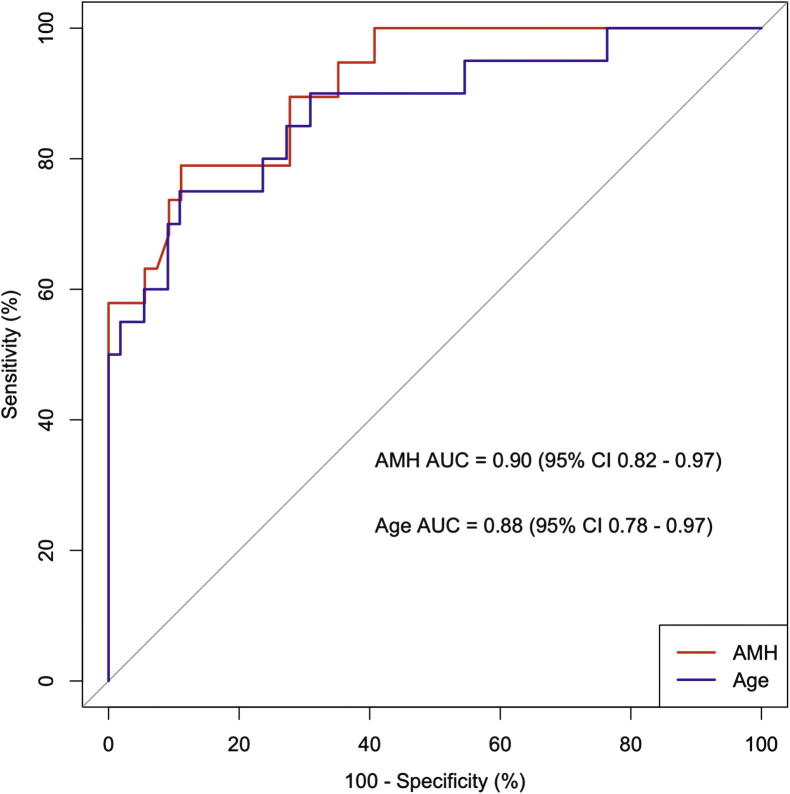
Receiver–operator characteristic (ROC) curve analysis of anti-Müllerian hormone (AMH) and age as predictors of ovarian function (indicated by ongoing menses) at 2 years (combined cohort: *n* = 75). Area under the curve for AMH (red):0.90 (95% confidence interval (CI) 0.82–0.97); for age (blue), 0.88 (95% CI 0.78–0.97).

Using the combined datasets (Table 3), the relative importance of age and AMH as predictors, calculated using Random Forests, showed that age was slightly less important than AMH (14.1 mean decrease in Gini index for age; 14.5 mean decrease in Gini index for AMH). The AUC of the ROC plot for AMH was 0.90 (95% CI 0.82 – 0.97)); the AUC of the ROC plot forage was 0.88 (95% CI 0.78–0.97) (Fig. 4), again indicating that both variables are important, with AMH slightly more important than age.

**Fig. 5:**
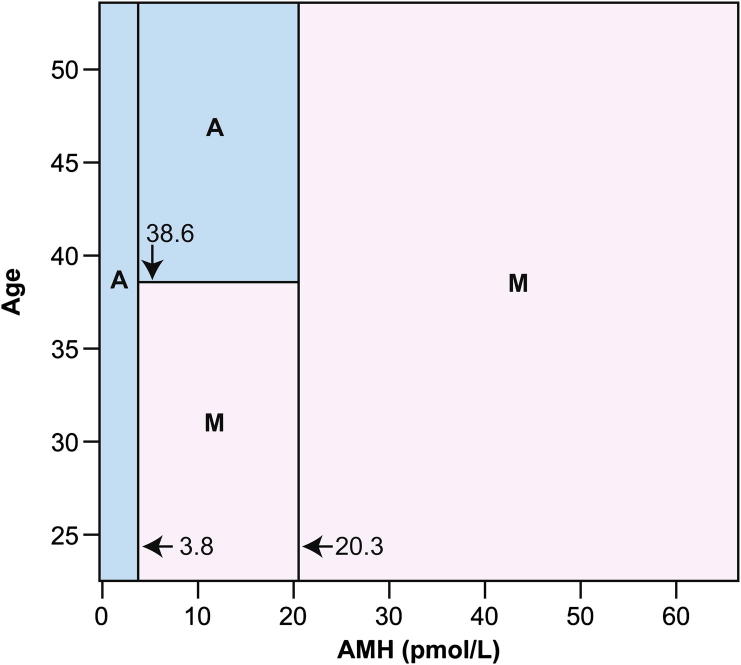
Classification mosaic chart for ongoing menses (M) or chemotherapy-related amenorrhoea (A) using serum anti-Müllerian hormone (AMH) and chronological age as predictor variables. The primary cutoff values are both for AMH, with below 3.8 pmol/L predicting amenorrhoea and above 20.3 pmol/L predicting ongoing menses. Between these AMH levels there is an age threshold at 38.6 years, above which amenorrhoea is predicted and below which ongoing menses are predicted. The classification schema has sensitivity 98.2% (one of 55 subjects known to have developed amenorrhoea misclassified as having ongoing menses) and specificity 80.0% (four of 20 subjects with known ongoing menses misclassified as amenorrhoeic). After 10-fold cross-validation this schema represents the optimal compromise between good fit to the data used to construct it, and low estimated error when used as a predictive model.

This secondary analysis indicated that predictive models derived using either age or AMH alone would be inferior to predictive models that incorporated both factors. We therefore derived a classification mosaic chart, shown in Fig. 5. This binary classification schema has sensitivity 98.2% and specificity 80.0%. The classification schema can be summarised as a division of subjects into three classes based on pretreatment AMH: low AMH subjects are classified as likely to develop amenorrhoea, and high AMH subjects are classified as likely to have ongoing menses. The medium AMH group is split into two classes at age 38.6 years; above this age threshold predicts amenorrhoea, and below predicts ongoing menses. If the clinical context requires that sensitivity be maximised, then the classification schema can be simplified to the initial split on AMH level, again using 20.3 pmol/L as the cutoff. In this case sensitivity is 100%, but specificity falls to 55%.

## 4 Discussion

The risk of ovarian failure following chemotherapy has previously been best predicted by the woman’s age.^3^ Prospective data show substantial differences in the prevalence of amenorrhoea in women with breast cancer, with 70% of women aged 40 and over having amenorrhoea after chemotherapy versus only 10% of those under 35,^4^ with comparable data provided by many similar studies. For some women, loss of ovarian function with chemotherapy is a concern, particularly when the benefit of the therapy may be modest. There are also emerging therapies with lower rates of amenorrhoea, though their efficacy remains unclear^31^ The ability to predict more accurately that risk for an individual woman is of increasing importance as their chances of survival continue to improve and with societal changes in age at childbirth. This will impact on the need to pursue fertility preservation strategies in some cases,^33,34^ and may influence decisions on treatment regimens.

The data presented here support the value of pretreatment measurement of AMH, but not other hormonal markers of the ovarian reserve, as an individualised predictor of the risk of amenorrhoea following chemotherapy for eBC. This study thus confirms and validates our previous similar findings^27^ and we have combined the two datasets to provide a classification mosaic. Our data confirm that age is a valuable predictor of ovarian function after chemotherapy for eBC: as an individual predictor, it performs very well (using a cut-off of 38.6 years). The key change in ovarian function with age is the steady decline in the size of the non-growing follicle pool, with the menopause occurring when the pool falls below a threshold to be able to support sufficient growing follicles to result in regular ovulation.^10^ Thus accurate measurement of the follicle pool is the key to assessment of individualisation of the impact of chemotherapy on the ovary. Serum AMH reflects both non-growing and growing ovarian follicle pools^15^ and declines with age.^35^ AMH predicts both time to, and age at, natural menopause,^14^ with age an important covariate. Several studies have shown a reduction in AMH in some childhood cancer survivors and following cancer treatment in adulthood,^18,21,23,24,36–38^ three studies have addressed the question of whether pretreatment AMH and other markers of the ovarian reserve can predict post-chemotherapy ovarian failure. We found that AMH, but not inhibin B, predicted long-term (4–5 year) ovarian function in women with early breast cancer,^27^ and others found that both AMH and inhibin B were lower in women with chemotherapy-associated amenorrhoea (CRA) at 1 year after chemotherapy for early breast cancer.^26^ However in a smaller study pre-chemotherapy AMH did not differ between those women who did or did not develop CRA,^28^ although the ascertainment of menses in that study was very limited.

The present data confirm our previous finding that in women with eBC, AMH is lower before treatment (approximately fourfold on average) in women who developed amenorrhoea after chemotherapy. Other biochemical markers of the ovarian reserve (FSH and inhibin B) showed no such predictive ability. Women with amenorrhoea were also older although regression analysis (including age, FSH and inhibin Bas recognised markers of the ovarian reserve) showed that at 2 years, only AMH was significantly and independently related to amenorrhoea. In the combined dataset, both AMH and age were confirmed to be predictive, with AMH slightly more so. Overall four different analytic methods confirmed the value of AMH with three also confirming the value of age, and from this we developed a classification mosaic. This was optimised to maximise both sensitivity and specificity, but can be adapted to the clinical scenario e.g. whether maximal sensitivity or specificity is the most important outcome.

A strength of this study is its prospective design, thus avoiding recall bias with careful ascertainment of menstrual function. A limitation is that all patients had eBC, thus its generalisability to other diseases and treatments is unclear, and the number of women included is small. While several treatment regimens were used to treat the women in this study, almost all included cyclophosphamide, recognised to be among the most gonadotoxic of therapies in women.^5^ The value of AMH in predicting post-cancer treatment ovarian function remains to be clearly demonstrated in younger women, although it can be used during and following treatment in children and adolescents.^39^

## 5 Conclusion

These data clearly confirm that women with a lower pretreatment AMH are more likely to develop amenorrhoea after chemotherapy for eBC. Thus measurement of AMH pretreatment may guide clinicians and women in treatment decisions and whether or not to consider fertility preservation strategies prior to treatment.

## Conflict of interest statement

Beckman Coulter provided some of the assay reagents used in this study. R.A. Anderson has undertaken consultancy work for Beckman Coulter and Roche Diagnostics. D.A. Cameron has received research funding unrelated to this work from Roche Diagnostics and Roche, and has undertaken unrelated consultancy work for Roche.

## Role of the funding source

The funder had no role in study design, analysis or decision to publish.

## Author contributions

R.A.A.: study design, data collection, analysis, drafting and finalising manuscript; M.R.: data collection, drafting and finalising manuscript; T.W.K.: data analysis, drafting and finalising manuscript; D.A.C.: study design, data analysis, drafting and finalising manuscript.

## Figures and Tables

**Table 1:** Details of chemotherapy regimens.

Regimen	Component drugs	No. of women	Duration (weeks)	Cyclophosphamide regimen	Cycles of taxane
*Non-trial*					
FEC	5FU, epiribucin + cyclophosphamide	4	18	3000 mg/m^2^ over 18 weeks	0
FEC-T	FEC followed by docetaxel	26	18	1500 mg/m^2^ over 9 weeks	3
E-CMF	Epirubicin q 21 d followed by CMF	16	24	3000 mg/m^2^ over 12 weeks	0
EC-T	Epirubicin + cyclophosphamide followed by docetaxol	6	18	1800 mg/m^2^ over 18 weeks	3
TC	Docetaxel with cylophosphamide	1	18	3600 mg/m^2^ over 18 weeks	6

*TACT2 trial*					
E-cCMF (TACT2)	Epirubicin q 21 d followed by CMF	3	28	4800 mg/m^2^ over 16 weeks	0
E-CAP	Epirubicin q 21 d followed by capecitabine	2	24	0	0
Accelerated E-cCMF	Epirubicin q 14 d followed by CMF	3	24	4800 mg/m^2^ over 16 weeks	0

cCMF: classical Bonnadona. CMF: cyclophosphamide, methotrexate, 5 fluorouracil.

**Table 2:** Pretreatment age and ovarian reserve markers by amenorrhoea/ongoing menses at 1 and 2 years.

	Amenorrhoea	Ongoing menses	*P*
*At 1 year*			
Age (years)	43.3 ± 0.7	37.9 ± 0.8	0.03
Anti-Müllerian hormone (AMH) (pmol/L)	6.6 ± 1.5	16.6 ± 4.8	0.01
Follicle-stimulating hormone (FSH) (IU/L)	4.9 ± 0.6	3.6 ± 0.9	ns
Inhibin B (pg/ml)	37.6 ± 5.8	32.4 ± 12.0	ns

*At 2 years*			
Age (years)	43.9 ± 0.8	37.9 ± 2.0	0.004
AMH (pmol/L)	4.0 ± 0.9	17.2 ± 5.1	<0.0001
FSH (IU/L)	5.6 ± 0.8	3.3 ± 0.5	ns
Inhibin B (pg/ml)	34.2 ± 6.2	38.1 ± 14.6	ns

**Table 3:** Demographic details of combined cohort (*n* = 75).

Age (year)	42.8 ± 0.7
Ethnicity (*n*)	73 Caucasian, one Asian, one Hispanic
Age at menarche (year)	13.0 ± 0.2
Previous pregnancies (*n*)	
None	14
First trimester only	2
Live birth	59
Current smoker (*n*)	15
Weight (kg)	69.0 ± 1.7
